# Opportunistic fungal pathogen *Candida glabrata* circulates between humans and yellow-legged gulls

**DOI:** 10.1038/srep36157

**Published:** 2016-10-26

**Authors:** Mohammed Hashim Al-Yasiri, Anne-Cécile Normand, Coralie L’Ollivier, Laurence Lachaud, Nathalie Bourgeois, Stanislas Rebaudet, Renaud Piarroux, Jean-François Mauffrey, Stéphane Ranque

**Affiliations:** 1 Aix-Marseille Univ, IP-TPT UMR MD3, 13885 Marseille, France; 2 APHM, CHU Timone, Laboratory of Parasitology - Mycology, 13005 Marseille, France; 3 CHU de Montpellier, Département de Parasitologie-Mycologie, Montpellier, France; 4 Faculté de Médecine de Montpellier-Nîmes, Nîmes, France; 5 Aix-Marseille Univ, IRD, Laboratoire Population Environnement Développement (LPED), UMR-151, Marseille, France

## Abstract

The opportunistic pathogenic yeast *Candida glabrata* is a component of the mycobiota of both humans and yellow-legged gulls that is prone to develop fluconazole resistance. Whether gulls are a reservoir of the yeast and facilitate the dissemination of human *C*. *glabrata* strains remains an open question. In this study, MLVA genotyping highlighted the lack of genetic structure of 190 *C*. *glabrata* strains isolated from either patients in three hospitals or fecal samples collected from gull breeding colonies located in five distinct areas along the French Mediterranean littoral. Fluconazole-resistant isolates were evenly distributed between both gull and human populations. These findings demonstrate that gulls are a reservoir of this species and facilitate the diffusion of *C*. *glabrata* and indirect transmission to human or animal hosts via environmental contamination. This eco-epidemiological view, which can be applied to other vertebrate host species, broadens our perspective regarding the reservoirs and dissemination patterns of antifungal-resistant human pathogenic yeast.

*Candida glabrata* is one of the most frequently identified yeast species of the human gut mycobiota^1^. This species has also emerged as a major agent of human mucosal, systemic and bloodstream yeast infections, second only to *C*. *albicans*^2^,^3^,^4^. *C*. *glabrata* infections are characterized by a high (40–70%) fatality rate, especially in immunocompromised patients^5^. However, the reservoir of *C*. *glabrata* has not been well characterized. Apart from humans, this yeast is a common commensal organism of many species of pet birds, such as cockatiels, parakeets, lovebirds and cockatoos or migratory birds such as the common whitethroat (*Sylvia communis*) and the spotted flycatcher (*Muscicapa striata*)^6^,^7^,^8^,^9^. A growing number of studies has demonstrated that birds act as a transporter and facilitate the spread of many pathogens, including viruses, bacteria, fungi and parasites^8^,^10^,^11^,^12^,^13^,^14^. Several pathogenic microorganisms have been shown to be transmitted over vast distances between humans and birds, such as the aquatic bird-borne influenza virus^15^. Studies have highlighted the risk of spreading various antibiotic resistant bacteria via contaminated bird feces^16^,^17^,^18^.

In recent decades, yellow-legged gull (*Larus michahellis*) population numbers have greatly increased throughout the Mediterranean littoral concurrent with an increase in anthropic refuse production. Consequently, they have displayed an increase in interaction with the environment in general and in particular with humans and other animals^19^,^20^,^21^,^22^. These birds feed on maritime or terrestrial matter, including human refuse, as demonstrated by an analysis of the composition of yellow-legged gull pellets in the Marseille region^23^.

Among pathogenic yeasts, *C*. *glabrata* is particularly prone to develop resistance to antifungal drugs, in particular fluconazole, which is the first-line antifungal treatment for yeast infections^24^. The extended use of fluconazole treatment in humans has been associated with an increase in fluconazole-resistant *C*. *glabrata* clinical isolates^25^,^26^,^27^. Yellow-legged gulls are known to be a reservoir of antibacterial-resistant Enterobacteriaceae (recently reviewed by Stedt *et al*.^28^). In a previous study, we have shown that *C*. *glabrata* constitutes part of the gut mycobiota of yellow-legged gulls^29^. However, whether yellow-legged gulls act as a reservoir and facilitate the dissemination of *C*. *glabrata* remains to be determined. Moreover, no data is available concerning the presence of antifungal-resistant yeast within the gut mycobiota of these birds. This study therefore aimed to assess whether *C*. *glabrata* isolates derived from sympatric yellow-legged gull and human populations are genetically distinct. We also determined whether fluconazole-resistant *C*. *glabrata* strains are present in the gut mycobiota of these birds.

## Results

MLVA revealed high genetic diversity with 129 distinct genotypes among the 190 *C*. *glabrata* isolates (Table 1). Allelic richness, diversity and evenness for each study site are detailed in Table 2. We found 100 singleton genotypes, of which 44 were isolated from humans and 56 were derived from gulls. Among the 29 non-singleton genotypes, 13 were present in both human and gull isolates, while 4 and 12 were only isolated in humans or gulls, respectively (Table 1).

**Figure 1:**
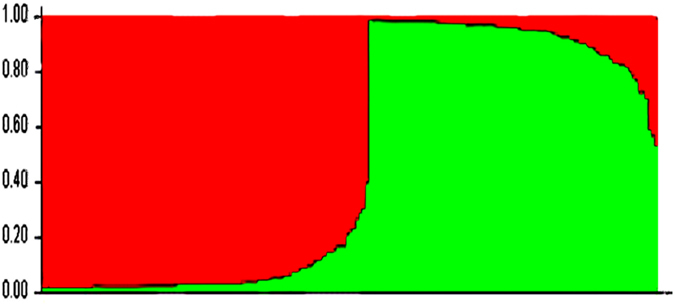
STRUCTURE clustering (admixture) in which each isolate is represented by a single vertical line that is partitioned into K = 2 colored segments. The segment length represents the individual’s estimated membership fractions in cluster 1 (red) and cluster 2 (green). Isolates with multiple colors have admixed genotypes from each cluster.

**Figure 2:**
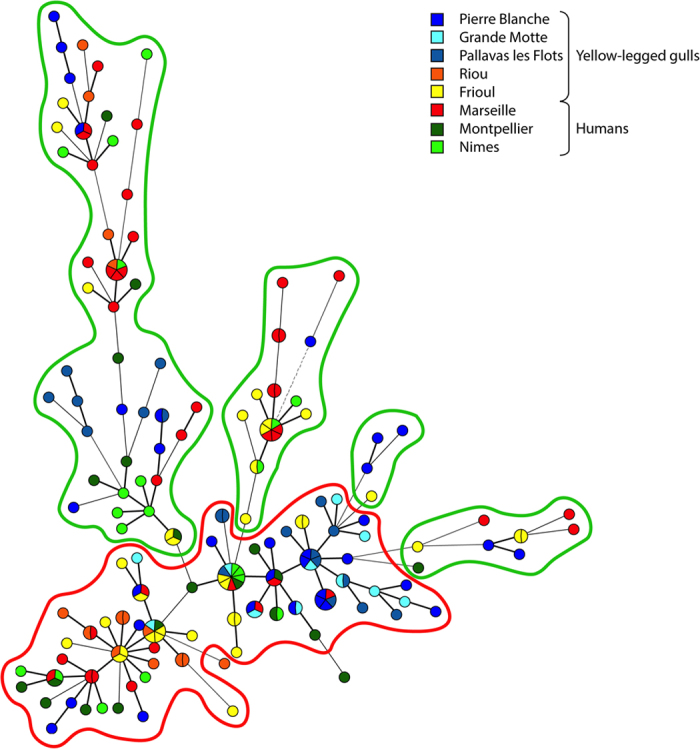
Minimum spanning tree of the 190 *C*. *glabrata* isolates collected from gulls or patients. Each node represents a unique MLVA genotype, and the various colors of the nodes indicate the study site. The single-locus variants are linked with thick solid lines, double-locus with thin solid line, while the triple-locus variants are linked with dashed lines. The two genetic clusters identified using STRUCTURE software are outlined in red (cluster 1; n = 101) and green (cluster 2; n = 89).

**Figure 3:**
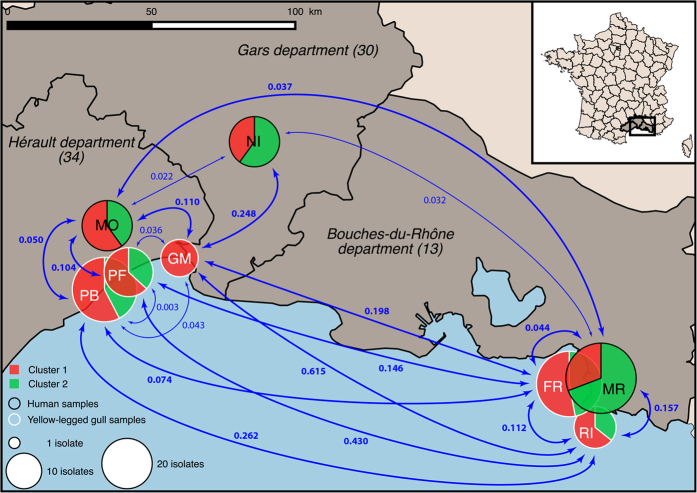
Map of the three cities, Montpellier, Nîmes and Marseille, and the yellow-legged gulls’ breeding colonies in the Mediterranean areas where isolates were sampled. The nodes indicate the relative number of isolates sampled in the respective region. Pie chart colors correspond to the proportion of two genetic clusters of the 190 *Candida glabrata* isolates at each site. *C*. *glabrata* population differentiation between study sites was measured via calculation of pairwise *F*_*ST*_. Statistically significant *F*_*ST*_ values are indicated in bold. The locations of the yellow-legged gulls’ breeding colonies are abbreviated as: PB = lagoon of Pierre Blanche; PF = Palavas-les-Flots; GM = La Grande-Motte; FR = Frioul Archipelago; and RI = Riou Archipelago). [*This map was created on the open source QGIS Geographic Information System software version 2*.*12*.*1-Lyon* (http://qgis.osgeo.org), *using an open license shapefile of French departments obtained from IGN* (http://professionnels.ign.fr/geofla)].

Two distinct genetic clusters, including 101 and 89 isolates, were identified by estimating the individual membership coefficient for each cluster using STRUCTURE (Fig. 1). The between-cluster *F*_*ST*_ was 0.27 (*P* < 10^−5^), thereby indicating, as expected, a significant effect of genetic structure on population differentiation. In contrast, the between-host *F*_*ST*_ was 0.03 (*P* < 10^−5^), which indicates a relatively low *C*. *glabrata* population differentiation due to genetic structure between gull and human isolates. The MST (Fig. 2) highlighted the high genetic diversity of *C*. *glabrata* isolates. Neither host (gull or human) nor study site was associated with any genetic cluster. In particular, gull and human isolates were distributed between both genetic clusters, with an exception for the 11 isolates collected from gulls at La Grande-Motte, which all belonged to the first cluster (Fig. 3).

AMOVA revealed that study sites, within-host and among-host populations explained 90% (*P* < 10^−5^), 9% (*P* < 10^−5^) and 1% (*P* = 0.11) of the genetic variance, respectively. We also calculated *F*_*ST*_ for three patient populations and five gull populations; the between site pairwise *F*_*ST*_ values are specified in Fig. 3 and Table 3. The population differentiation due to genetic structure between sites (hospitals) of human isolates was relatively low; the highest of these *F*_*ST*_ values was 0.037 (*P* = 0.04) between populations from Montpellier and Marseille (Fig. 3, Table 3). The population differentiation due to genetic structure between sites (breeding colonies) of yellow-legged gull isolates was comparatively higher than among the human isolates. In particular, differentiation due to genetic structure was particularly high between the population from the Riou Archipelago and those from all other study sites. The overall highest pairwise *F*_*ST*_ value was 0.615 (*P* < 0.001) between Riou and La Grande-Motte (Fig. 3, Table 3). The Mantel test showed that geographical distances explained 14.4% of *C*. *glabrata* genetic differentiation (*P* = 0.023).

**Figure 4:**
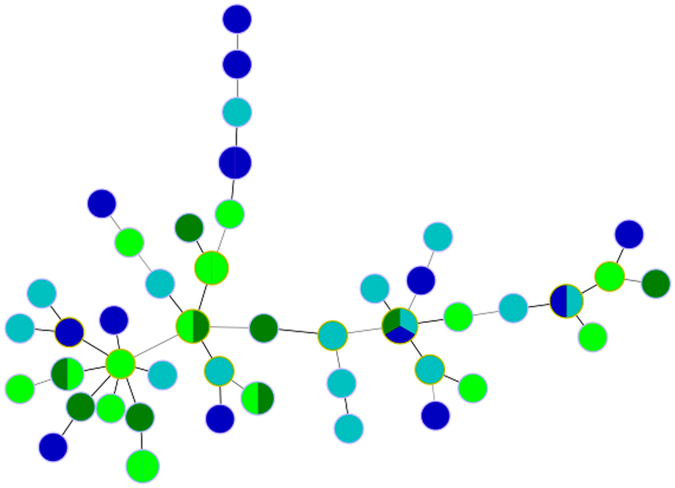
MLVA-based minimum spanning tree of 54 *C*. *glabrata* isolates from Marseille hospital patients and gulls from the Riou and Frioul archipelagos off the coast of Marseille. *In vitro* resistance to the antifungal fluconazole was found in 9 and 14 isolates from gull and human hosts, respectively. Blue nodes indicate human isolates; green nodes indicate gull isolates; dark colors indicate resistance to fluconazole and light colors indicate susceptibility to fluconazole.

*In vitro* fluconazole susceptibility was assessed in 54 *C*. *glabrata* isolates, all of which were collected within the same time period in the Marseille area, including 25 samples collected from yellow-legged gull breeding colonies on the Frioul and Riou Archipelagos and 29 isolates collected from patients at the university hospital of Marseille. Overall, 23 isolates were classified as fluconazole resistant (minimal inhibitory concentration ≥64 mg/L); 9 and 14 (36.5%, 95% confidence interval (CI) [18.0–57.5%] vs. 50%, 95%CI [29.5–67.5%], *P* = 0.53) were isolated from gull or human hosts, respectively. The absence of genetic clustering according to the host or fluconazole susceptibility is depicted in the MLVA-based MST tree (Fig. 4).

## Discussion

Overall, this study highlights the absence of significant genetic differentiation between *C*. *glabrata* populations in humans or yellow-legged gulls. We also demonstrated that antifungal-resistant isolates are present within the gut mycobiota of yellow-legged gulls. The low differentiation between human and gull *C*. *glabrata* populations is in agreement with a previous study that has shown that *C*. *dubliniensis* populations isolated from herring gulls (*Larus argentatus*) or humans were genetically similar^30^. In contrast, geographic location of the collection site was the major factor in genetic variance. Furthermore, Mantel test analysis showed a trend of increasing genetic differentiation with increasing geographical distance. Similarly, de Meeûs *et al*.^31^ have shown using both multilocus enzyme electrophoresis and randomly amplified polymorphic DNA that *C*. *glabrata* populations isolated from patients in Paris and Montpellier, which are 800 km apart, displayed genetic differentiation (*F*_*ST*_ = 0.11, *P* = 0.054). Using the same MLVA scheme as in the present study, Dhieb *et al*.^32^ have shown highly significant genetic differentiation (*F*_*ST*_ = 0.359, *P* < 10^−5^) between *C*. *glabrata* populations isolated from patients in France or Tunisia. Although the geographical scale of our study was much more limited than in the previous studies, we detected either relatively high or low genetic differentiation according to the study sites. As we found evidence for both dispersion and differentiation, our study was indeed adequately scaled to dissect transmission profiles and detect reservoirs.

*C*. *glabrata* is a component of the human gut mycobiota. We hypothesize that yellow-legged gulls inadvertently ingest yeast such as *C*. *glabrata* with their food, which might be contaminated with human excreta in highly anthropic marine and terrestrial environments. Compared with other sea birds, yellow-legged gulls are highly synanthropic. This is a major reason that gull populations have grown concomitantly with human-made environments, including human refuse sites, along the Mediterranean littoral^33^. Moreover, beach sand may also play a role as a reservoir for *C*. *glabr*ata^34^. The birds become a reservoir as the yeast develops into a component of the gut microbiota. The yellow-legged gull can fly relatively extended distances along the Mediterranean littoral to feed on landfills. Therefore, garbage dumps in urbanized areas may be a potential source of clinically important yeast transmitted by gulls. Due to the high mobility of gulls, the birds facilitate the dissemination of the yeast by releasing their droppings over an expansive area of the marine and terrestrial environment.

Humans may be infected with *C*. *glabrata* originating directly (via bird droppings in their direct environment) or, more frequently, indirectly by ingesting food that has been contaminated with bird droppings. The genetic homogeny of human and bird isolates clearly suggests that yellow-legged gulls play a role in the diffusion of *C*. *glabrata* acquired from an anthropic environment. In line with this hypothesis, yellow-legged gulls transmit and spread potential human pathogens in various environments. Indeed, Bonnedahl *et al*.^16^ have demonstrated that yellow-legged gulls disseminate antibiotic-resistant *Escherichia coli* isolates not far from Pierre Blanche. Similarly, we demonstrated the presence of genetically homogeneous fluconazole-resistant *C*. *glabrata* populations in both gulls and humans in Marseille. Therefore, our findings show that yellow-legged gulls act as a carrier, reservoir and disseminator of *C*. *glabrata*. These birds may thus contribute to the transmission of yeast to humans and likely other animal hosts. Moreover, the likelihood of infectious disease transmission via yellow-legged gulls has therefore increased due to the marked demographic growth of gull populations along the Mediterranean littoral. Our findings also demonstrate that yellow-legged gulls represent a reservoir of antifungal-resistant *Candida* strains and disseminate antifungal-resistant isolates.

## Conclusions

The close proximity and interaction between very dense human and yellow-legged gull populations in cities of the Mediterranean littoral facilitates the circulation of microorganisms between the two hosts. Gulls likely ingest *C*. *glabrata* by eating or drinking in environments contaminated with human excreta. The yeast eventually evolves to be incorporated into their gut mycobiota and is spread via gull feces to human environment. Moreover, yellow-legged gulls represent a reservoir of fluconazole-resistant *C*. *glabrata* isolates, which may have a potential impact on public health, including food spoilage control and potable water source protection.

## Methods

### C. glabrata isolates

In this study, we analyzed 190 *C*. *glabrata* isolates. One hundred eleven samples were isolated from feces collected on the soil at five yellow-legged gull breeding colonies as previously described by Al-Yasiri *et al*.^29^. Briefly, the sampled breeding colonies were located in the departments of Hérault and Bouches-du-Rhône, in the South of France. In Hérault, the colonies were located in a natural reserve at the lagoon of Pierre Blanche and in two cities, Palavas-les-Flots and La Grande-Motte. In Bouches-du-Rhône, two breeding colonies were located on the Frioul and Riou Archipelagos off the coast of Marseille. Yellow-legged gulls may yet be exposed to varying levels of anthropogenic pressure. In this study for instance, birds breeding at the lagoon of Pierre Blanche were exposed to a relatively low anthropogenic influence compared with those breeding on the building rooftops in the cities of Palavas-les-Flots, La Grande-Motte and Marseille. Riou and Frioul Archipelagos are suburban ecocline exposed to an intermediate anthropogenic influence. Seventy-nine *C*. *glabrata* samples were isolated from patients at three university hospitals in Marseille, Montpellier and Nimes, which were located in the same geographical area and sampled during the same time period as those isolated from gulls. All isolates were subcultured on Malt extract agar (Sigma Aldrich, USA). The samples were identified via the MALDI-TOF MS (matrix-assisted laser desorption/ionization-time-of-flight mass spectrometry) technique, as previously described^35^, and subcultured onto chromogenic medium plates (CHROMagar^TM^, Becton Dickinson, France) to verify isolate purity^36^.

### Multiple-locus variable number tandem repeat analysis (MLVA)

All *C*. *glabrata* isolates were typed with eight microsatellite markers (GLA2, GLA3, GLA4, GLA5, GLA6, GLA7, GLA8 and GLA9) as previously described by Brisse *et al*.^37^. Genomic DNA was extracted using the NucliSENS^TM^ EasyMAG^TM^ (bioMérieux) system^38^, eluted in 50 μl and stored at −20 °C. Amplification reactions were performed using a Lightcycler^TM^ 480 (Roche Diagnostics, GmbH, Germany) instrument with Lightcycler^TM^ 480 Probes Master (Roche Diagnostics, GmbH, Germany). The loci, primer sequences, fluorophores and hybridization temperatures are described in Table 4. The PCR products were visualized using 2% agarose gel electrophoresis in 1X of Tris borate EDTA buffer (Euromedex, France) with SYBR^TM^ safe DNA gel stain (Invitrogen, USA). Next, 1 μl of 1:100 diluted PCR products was mixed with a solution containing 25 μl HiDi formamide (Life Technologies, France) and 0.5 μl Gene Scan^TM^ 500 LIZ^TM^ size standard (Applied Biosystems, UK). The fragment length was determined via capillary electrophoresis using an ABI 3130 Genetic Analyzer (Applied Biosystems, France) and analyzed using GeneMapper software v4.0 (Applied Biosystems, France).

### Population genetic analysis

Several indices of clonal diversity were estimated using the *poppr* R package^39^, including the genetic richness, i.e. the number of multilocus genotypes (MLG) observed per population and the number of expected MLG at the smallest sample size based on rarefaction (eMLG); the genetic evenness *E*_*5*_, (ranging from 0, a population composed of a single genotype, to 1, all genotypes having equal frequency); Simpson’s (lambda) diversity index^40^ corrected by the number of isolates in a population; and the clonal fraction^41^.

STRUCTURE v. 2.3.1 was used to identify genetically distinct clusters via estimation of the proportion of membership (Q) of each isolate in each cluster. This software applies a Bayesian model-based clustering approach^42^ to model K populations that are characterized by a set of allele frequencies at each locus and probabilistically assigns isolates to clusters based on the multilocus genotypes. For each K value, ranging from 1 to 10, the posterior probability of the data was measured using an admixture model with correlated allele frequencies over 10 independent runs with a burn-in period of 5000 followed by 50000 Markov Chain Monte Carlo steps. The number of clusters was estimated by the value of K that maximizes the posterior probability of the data. The highest ΔK value corresponds to the most pronounced partition of the data and indicates the likely number of clusters.

The among- and between-population differentiation due to genetic structure was estimated via Slatkin’s linearized pairwise fixation index (*F*_*ST*_) and Analysis of MOlecular VAriance (AMOVA) using ARLEQUIN v3.5 software. The effect of geographical distances on genetic differentiation was tested via Mantel test. MLVA-based Minimum Spanning Tree (MST) was constructed using BIONUMERICS software v7.1.

### Fluconazole susceptibility testing

*C*. *glabrata* anti-fluconazole susceptibility was assessed as described by Clinical Laboratory Standards Institute (CLSI) M27-S4 document^43^. Fluconazole resistance was defined as a minimal inhibitory concentration of ≥64 mg/L. Each assay was validated using ATCC 22019 (*Candida parapsilosis*) and ATCC 6258 (*Candida krusei*) quality control strains.

## Additional Information

**How to cite this article**: Al-Yasiri, M. H. *et al*. Opportunistic fungal pathogen *Candida glabrata* circulates between humans and yellow-legged gulls. *Sci. Rep.* **6**, 36157; doi: 10.1038/srep36157 (2016).

**Publisher’s note:** Springer Nature remains neutral with regard to jurisdictional claims in published maps and institutional affiliations.

## Figures and Tables

**Table 1:** Frequency distribution of the 129 MLVA genotypes identified for the 190 *C*. *glabrata* samples isolated from yellow-legged gulls or humans.

Genotypes	Gull	Human	Total
65	4	1	5
66	4	5	9
68	5	2	7
98	1	4	5
121	2	4	6
17	2	1	3
42	1	2	3
43	2	1	3
70	2	1	3
77	1	2	3
24	1	1	2
86	1	1	2
124	1	1	2
102	3	0	3
41	5	0	5
27	2	0	2
39	2	0	2
40	2	0	2
57	2	0	2
67	2	0	2
75	2	0	2
100	2	0	2
101	2	0	2
105	2	0	2
122	2	0	2
3	0	3	3
88	0	2	2
112	0	2	2
113	0	2	2
Singletons*	56	44	100
Total	**111**	**79**	**190**

*A singleton is a genotype that has been found only once in the study population.

**Table 2:** Allelic richness, diversity and evenness of the 111 MLVA genotypes identified for the 190 *C*. *glabrata* samples isolated from yellow-legged gulls or humans were estimated for each study site.

Study site	N	MLG	eMLG	E_5_	Corrected lambda	Clonal fraction
Pierre Blanche	33	30	10.61	0.93	0.99	0.09
La Grande-Motte	11	11	11.00	1.00	1.00	0.00
Palavas- les-Flots	19	17	10.36	0.95	0.99	0.11
Riou	14	12	9.79	0.94	0.98	0.14
Frioul	34	25	10.05	0.92	0.98	0.26
Marseille	39	31	10.29	0.90	0.99	0.21
Montpellier	20	20	11.00	1.00	1.00	0.00
Nîmes	20	18	10.28	0.90	0.98	0.10
Total	**190**	**131**	**10.62**	**0.74**	**0.99**	**0.31**

Allelic richness was specified using the number of multilocus genotypes observed per population (MLG) and the number of expected MLG at the smallest sample size based on rarefaction (eMLG). Genetic evenness was estimated using the E5 index. Diversity was calculated using the Simpson’s (lambda) index corrected by the number of isolates in a population.

**Table 3:** Pairwise *C*. *glabrata* population differentiation matrix according to the study sites.

	PB	PF	GM	RI	FR	MA	MO	NI
PB		0.35	0.08	<0.001	<0.001	<0.001	0.01	<0.001
PF	0.003		0.16	<0.001	<0.001	<0.001	0.001	<0.001
GM	0.043	0.036		<0.001	<0.001	<0.001	0.009	<0.001
RI	**0.262**	**0.43**	**0.615**		<0.002	<0.001	<0.001	<0.001
FR	**0.074**	**0.146**	**0.198**	**0.112**		0.009	0.012	0.06
MA	**0.071**	**0.132**	**0.215**	**0.157**	**0.044**		0.04	0.06
MO	**0.050**	**0.104**	**0.11**	**0.229**	**0.052**	**0.037**		0.17
NI	**0.085**	**0.139**	**0.248**	**0.205**	0.035	0.032	0.022	

Slatkin’s linearized *F*_*ST*_ fixation index values are tabulated in the lower triangle, and the corresponding *P* values are indicated in the upper triangle. Statistically significant *F*_*ST*_ values is indicated in bold text.

Study site abbreviations. Yellow-legged gulls’ breeding colonies: PB = lagoon of Pierre Blanche; PF = Palavas-les-Flots; GM = La Grande-Motte; RI = Riou Archipelago and FR = Frioul Archipelago. University hospitals of MA = Marseille, MO = Montpellier and NI = Nîmes.

**Table 4:** Characteristics of the eight microsatellite loci used for *C*. *glabrata* MLVA genotyping.

locus	Chromosome	Gene or intergene	repeat in CBS138	Forward primer	Reverse primer	Temperature of hybridization
GLA2	I	Intergene	(GCT)_8_	6-FAM-GCACTCTGTCTACTTATAC	CGAATCCGTGATCCCTTC	54
GLA3	D	Intergene	(AAC)_6_	6-FAM-ACACCTACGAGAAACCAACA	TAGCGGTCATCCAGCATCA	56
GLA4	A	Intergene	(ATC)_8_	VIC-ATGCTGTATTTAACGATGCC	ACCAATGGTAACAGAGT	54
GLA5	H	CAGL0H02783 g	(ATC)_10_	VIC-TTATTACTCTTTCGGGTCAGG	CGAAACAACGTCAGAAACTC	54
GLA6	J	CAGL0J10560 g	(ATC)_9_	VIC-CCTTTAAGGATGAGCTACTTC	GCTGGTGGTTTAAAGGAAAC	54
GLA7	K	CAGL0K05423 g	(GTT)_13_	PET-GTCGTACCTTTGTATAATGTTG	AATGCGTTAGATGCCTT GAGA	50
GLA8	C	CAGL0C01265 g	(TAA)_11_	PET-ATTTACTAATAATACAGCTCAC	CTCAGCAGAACTTTCTT TAGT	50
GLA9	I	Intergene	(ACTC)_5_	PET-CCTTCCCTCTGCGGATACT	TCACTGGACCTCTGTAG TGGT	56

## References

[b1] Khatib R., Riederer K. M., Ramanathan J. & Baran J. Faecal fungal flora in healthy volunteers and inpatients. Mycoses 44, 151–156 (2001). 11486452 10.1046/j.1439-0507.2001.00639.x

[b2] Falagas M. E., Roussos N. & Vardakas K. Z. Relative frequency of albicans and the various non-albicans *Candida* spp among candidemia isolates from inpatients in various parts of the world: a systematic review. Int. J. Infect. Dis. 14, e954–e966 (2010). 20797887 10.1016/j.ijid.2010.04.006

[b3] Silva S. . *Candida glabrata*, *Candida parapsilosis* and *Candida tropicalis*: biology, epidemiology, pathogenicity and antifungal resistance. FEMS Microbiol. Rev. 36, 288–305 (2012). 21569057 10.1111/j.1574-6976.2011.00278.x

[b4] Jacobsen I. D. . *Candida glabrata* Persistence in Mice Does Not Depend on Host Immunosuppression and Is Unaffected by Fungal Amino Acid Auxotrophy. Infect. Immun. 78, 1066–1077 (2010). 20008535 10.1128/IAI.01244-09 PMC2825948

[b5] Krcmery V. & Barnes A. J. Non-*albicans Candida* spp. causing fungaemia: pathogenicity and antifungal resistance. J. Hosp. Infect. 50, 243–260 (2002). 12014897 10.1053/jhin.2001.1151

[b6] Mancianti F., Nardoni S. & Ceccherelli R. Occurrence of yeasts in psittacines droppings from captive birds in Italy. Mycopathologia 153, 121–124 (2002). 11998871 10.1023/a:1014576304894

[b7] Brilhante R. S. N. . Characterization of the gastrointestinal yeast microbiota of cockatiels (*Nymphicus hollandicus*): a potential hazard to human health. J. Med. Microbiol. 59, 718–723 (2010). 20150318 10.1099/jmm.0.017426-0

[b8] Cafarchia C., Romito D., Coccioli C., Camarda A. & Otranto D. Phospholipase activity of yeasts from wild birds and possible implications for human disease. Med. Mycol. Off. Publ. Int. Soc. Hum. Anim. Mycol. 46, 429–434 (2008). 10.1080/13693780701885636 18608940

[b9] Francesca N. . Yeasts vectored by migratory birds collected in the Mediterranean island of Ustica and description of *Phaffomyces usticensis* f.a. sp. nov., a new species related to the cactus ecoclade. FEMS Yeast Res. 14, 910–921 (2014). 24981278 10.1111/1567-1364.12179

[b10] Webster R. G., Bean W. J., Gorman O. T., Chambers T. M. & Kawaoka Y. Evolution and Ecology of influenza A viruses. Microbiol. Rev. 56, 152–179 (1992). 1579108 10.1128/mr.56.1.152-179.1992 PMC372859

[b11] Fouchier R. A. M. . Characterization of a novel influenza A virus hemagglutinin subtype (H16) obtained from black-headed gulls. J. Virol. 79, 2814–2822 (2005). 15709000 10.1128/JVI.79.5.2814-2822.2005 PMC548452

[b12] Andersen A. A. Two new serovars of *Chlamydia psittaci* from North American birds. J. Vet. Diagn. Investig. Off. Publ. Am. Assoc. Vet. Lab. Diagn. Inc 9, 159–164 (1997). 10.1177/104063879700900209 9211235

[b13] Pattron D. D. Aspergillus, Health Implication & Recommendations for Public Health Food Safety. J. Food Saf. 8, 19–23 (2006).

[b14] Rinaldi L. & Scala A. Toxoplasmosis in livestock in Italy: an epidemiological update. Parassitologia 50, 59–61 (2008). 18693559

[b15] Fouchier R. A. M., Osterhaus A. D. M. E. & Brown I. H. Animal influenza virus surveillance. Vaccine 21, 1754–1757 (2003). 12686089 10.1016/s0264-410x(03)00067-7

[b16] Bonnedahl J. . Dissemination of *Escherichia coli* with CTX-M type ESBL between humans and yellow-legged gulls in the south of France. PloS One 4, e5958 (2009). 19536298 10.1371/journal.pone.0005958 PMC2694269

[b17] Lord A. T. K., Mohandas K., Somanath S. & Ambu S. Multidrug resistant yeasts in synanthropic wild birds. Ann. Clin. Microbiol. Antimicrob. 9, 1–5 (2010). 20307325 10.1186/1476-0711-9-11 PMC2852373

[b18] Horton R. A. . Fecal carriage and shedding density of CTX-M extended-spectrum {beta}-lactamase-producing *Escherichia coli* in cattle, chickens, and pigs: implications for environmental contamination and food production. Appl. Environ. Microbiol. 77, 3715–3719 (2011). 21478314 10.1128/AEM.02831-10 PMC3127594

[b19] Arcos J., Oro D. & Sol D. Competition between the yellow-legged gull *Larus cachinnans* and Audouin’s gull *Larus audouinii* associated with commercial fishing vessels: the influence of season and fishing fleet. Mar. Biol. 139, 807–816 (2001).

[b20] Duhem C., Roche P., Vidal E. & Tatoni T. Effects of anthropogenic food resources on yellow-legged gull colony size on Mediterranean islands. Popul. Ecol. 50, 91–100 (2008).

[b21] Ramos R., Ramírez F., Sanpera C., Jover L. & Ruiz X. Diet of yellow-legged Gull (*Larus michahellis*) chicks along the Spanish Western Mediterranean coast: the relevance of refuse dumps. J. Ornithol. 150, 265–272 (2008).

[b22] Ramos R., Ramírez F., Sanpera C., Jover L. & Ruiz X. Feeding ecology of yellow-legged gulls *Larus michahellis* in the western Mediterranean: a comparative assessment using conventional and isotopic methods. Mar. Ecol. Prog. Ser. 377, 289–297 (2009).

[b23] Duhem C., Vidal E., Legrand J. & Tatoni T. to accessibility of refuse dumps: The gulls adjust their diet composition and diversity according to refuse dump accessibility. Bird Study 50, 61–67 (2003).

[b24] Abbes S. . Microsatellite analysis and susceptibility to FCZ of *Candida glabrata* invasive isolates in Sfax Hospital, Tunisia. Med. Mycol. 49, 10–15 (2011). 20586679 10.3109/13693786.2010.493561

[b25] Pfaller M. A. . Geographic variation in the frequency of isolation and fluconazole and voriconazole susceptibilities of *Candida glabrata*: an assessment from the ARTEMIS DISK Global Antifungal Surveillance Program. Diagn. Microbiol. Infect. Dis. 67, 162–171 (2010). 20338711 10.1016/j.diagmicrobio.2010.01.002

[b26] Fidel P. L. Jr., Vazquez J. a & Sobel J. D. *Candida glabrata*: review of epidemiology, pathogenesis, and clinical disease with comparison to *C. albicans*. Clin Microbiol Rev 12, 80–96 (1999). 9880475 10.1128/cmr.12.1.80 PMC88907

[b27] Lott T. J., Frade J. P., Lyon G. M., Iqbal N. & Lockhart S. R. Bloodstream and non-invasive isolates of *Candida glabrata* have similar population structures and fluconazole susceptibilities. Med. Mycol. 50, 136–142 (2012). 21838617 10.3109/13693786.2011.592153

[b28] Stedt J., Bonnedahl J., Hernandez J. & Mcmahon B. J. Antibiotic resistance patterns in *Escherichia coli* from gulls in nine European countries. Infect. Ecol. Epidemiol. 1, 1–10 (2014). 10.3402/iee.v4.21565 PMC3889177 24427451

[b29] Al-Yasiri M. H., Normand A., Piarroux R., Ranque S. & Mauffrey J.-F. Yeast gut communities in various *Larus michahellis* breeding colonies. Med Mycol. in press, 10.1093/mmy/myw088. 27703020

[b30] McManus B. A. . Genetic differences between avian and human isolates of *Candida dubliniensis*. Emerg. Infect. Dis. 15, 1467–1470 (2009). 19788816 10.3201/eid1509.081660 PMC2819872

[b31] de Meeûs T. . Genetic Structure of *Candida glabrata* Populations in AIDS and Non-AIDS Patients. Journal of Clinical Microbiology, 40, 2199–2206 (2002). 12037087 10.1128/JCM.40.6.2199-2206.2002 PMC130676

[b32] Dhieb C. . MALDI-TOF typing highlights geographical and fluconazole resistance clusters in *Candida glabrata*. Med. Mycol. 53, 462–469 (2015). 25841053 10.1093/mmy/myv013

[b33] Neves V. C., Murdoch N. & Furness R. W. Population status and diet of the yellow-legged Gull in the Azores. Life and Marine Sciences 23A, 59–73 (2006).

[b34] Shah A. H. . Indicator microbes correlate with pathogenic bacteria, yeasts and helminthes in sand at a subtropical recreational beach site. J. Appl. Microbiol. 110, 1571–1583 (2011). 21447014 10.1111/j.1365-2672.2011.05013.x

[b35] Cassagne C. . Evaluation of four pretreatment procedures for MALDI-TOF MS yeast identification in the routine clinical laboratory. Med. Mycol. 51, 371–377 (2012). 22978312 10.3109/13693786.2012.720720

[b36] Cassagne C. . Routine identification and mixed species detection in 6,192 clinical yeast isolates. Med. Mycol. myv095, 10.1093/mmy/myv095 (2015). 26613703

[b37] Brisse S. . Uneven Distribution of Mating Types among Genotypes of *Candida glabrata* Isolates from Clinical Samples. Eukaryot. Cell 8, 287–295 (2009). 19151326 10.1128/EC.00215-08 PMC2653237

[b38] Jeddi F., Piarroux R. & Mary C. Application of the NucliSENS easyMAG system for nucleic acid extraction: optimization of DNA extraction for molecular diagnosis of parasitic and fungal diseases. Parasite Paris Fr. 20, 52 (2013). 10.1051/parasite/2013051 PMC3859032 24331004

[b39] Kamvar Z. N., Tabima J. F. & Grünwald N. J. Poppr: an R package for genetic analysis of populations with clonal, partially clonal, and/or sexual reproduction. PeerJ 2, e281 (2014). 24688859 10.7717/peerj.281 PMC3961149

[b40] Simpson E. H. Measurement of Diversity. Nature 163, 688–688 (1949).

[b41] Coletta-Filho H. D., Bittleston L. S. & Almeida R. P. P. Spatial genetic structure of a vector-borne generalist pathogen. Appl. Environ. Microbiol. 77, 2596–2601 (2011). 21317251 10.1128/AEM.02172-10 PMC3126377

[b42] Pritchard J. K., Stephens M. & Donnelly P. Inference of population structure using multilocus genotype data. Genetics 155, 945–959 (2000). 10835412 10.1093/genetics/155.2.945 PMC1461096

[b43] Clinical Laboratory Standards Institute. Reference Method for Broth Dilution Antifungal susceptibility testing of yeast. Clin. Lab. Stand. Inst. 32, 1–23 (2012).

